# Polyphenols as Multi-Target Regulators of Oxidative Stress, Mitochondrial Function, and Cell Survival Signaling in Skin Diseases

**DOI:** 10.3390/ijms27177877

**Published:** 2026-09-03

**Authors:** Moon-Kyun Cho, Min Hyuk Choi, Ki Dam Kim, Sukh Que Park, Sang-Han Lee, Hae-Seon Nam, Yoon-Jin Lee

**Affiliations:** 1 Division of Molecular Cancer Research, Soonchunhyang Medical Research Institute, Soonchunhyang University, Cheonan 31511, Republic of Korea; mkcho@schmc.ac.kr (M.-K.C.); m1037624@sch.ac.kr (S.-H.L.); namhs@sch.ac.kr (H.-S.N.); 2 Department of Dermatology, Soonchunhyang University Hospital, Seoul 04401, Republic of Korea; 146079@schmc.ac.kr (M.H.C.); 132351@schmc.ac.kr (K.D.K.); 3 Department of Neurosurgery, Soonchunhyang University Hospital, Seoul 04401, Republic of Korea; drcolor@schmc.ac.kr; 4 Department of Biochemistry, College of Medicine, Soonchunhyang University, Cheonan 31511, Republic of Korea; 5 Department of Tropical Medicine, College of Medicine, Soonchunhyang University, Cheonan 31511, Republic of Korea

**Keywords:** polyphenols, skin diseases, oxidative stress, mitochondrial function, Nrf2, NF-κB, PI3K/Akt, MAPK/ERK, AMPK, inflammation

## Abstract

Bioactive polyphenols have emerged as multi-target regulators of cellular processes involved in the pathogenesis of skin diseases. Skin disorders, including inflammatory conditions, photoaging, and skin cancers, are characterized by complex pathogenic mechanisms associated with oxidative stress, mitochondrial dysfunction, dysregulated signaling pathways, and metabolic imbalance. Excessive production of reactive oxygen species (ROS) and persistent inflammatory signaling contribute to disease progression and cellular adaptation under stress conditions. Unlike conventional agents that typically target a single pathway, polyphenols act on interconnected signaling and metabolic networks. These compounds regulate key signaling pathways, including phosphoinositide 3-kinase/protein kinase B (PI3K/Akt), mitogen-activated protein kinase/extracellular signal-regulated kinase (MAPK/ERK), AMP-activated protein kinase (AMPK), nuclear factor-κB (NF-κB), and nuclear factor erythroid 2-related factor 2 (Nrf2)**,** thereby regulating cell survival, proliferation, inflammatory responses, antioxidant defense, and metabolic adaptation. Polyphenols also influence mitochondrial function by maintaining redox homeostasis, regulating energy metabolism, and affecting apoptosis-related signaling pathways. This review provides a mechanistic overview of the effects of polyphenols on oxidative stress, mitochondrial function, and cell survival signaling in skin diseases. In addition, the therapeutic implications and current limitations of polyphenol-based approaches are discussed, with particular emphasis on the translational gap between experimental findings and physiological relevance. Factors such as concentration, bioavailability, and cellular microenvironment are highlighted as major determinants of polyphenol activity and key challenges for clinical translation. Finally, the need for further in vivo and clinical investigations is emphasized to support the development of effective polyphenol-based therapeutic strategies for skin diseases.

## 1. Introduction

Skin diseases comprise a diverse spectrum of inflammatory, degenerative, and malignant disorders that collectively impose a substantial global health burden and significantly reduce quality of life [1]. These conditions include chronic inflammatory diseases such as psoriasis and atopic dermatitis, as well as skin cancers including melanoma and non-melanoma skin cancers, which arise through complex interactions among genetic, environmental, and metabolic factors [2,3,4,5]. Despite differences in clinical presentation, many skin diseases share common pathogenic mechanisms, including oxidative stress, chronic inflammation, dysregulated cellular signaling, and impaired metabolic homeostasis [2,3,4,5,6].

Oxidative stress plays a central role in skin pathophysiology. Excessive production of reactive oxygen species (ROS), frequently induced by environmental stressors such as ultraviolet (UV) radiation, induces cellular damage, disrupts redox homeostasis, and activates inflammatory signaling pathways [6,7]. In addition to UV radiation, oxidative stress in the skin can be triggered or amplified by multiple pathological stimuli, including microbial infections, chronic inflammatory responses, and malignant transformation. These conditions can promote sustained ROS generation through inflammatory cell activation, mitochondrial dysfunction, and altered cellular metabolism, thereby further disrupting redox homeostasis and contributing to disease progression [6,8,9,10]. Persistent oxidative stress further contributes to disease initiation, progression, and adaptive cellular responses under chronic pathological conditions [6,11]. In addition to oxidative stress, mitochondrial dysfunction has emerged as an important contributor to the pathogenesis of skin diseases. Mitochondria play essential roles in cellular energy metabolism, redox regulation, and apoptosis, and mitochondrial impairment can disrupt metabolic activity, increase ROS production, and impair cellular stress adaptation [12].

These alterations are closely associated with dysregulation of key signaling pathways, including phosphoinositide 3-kinase/protein kinase B (PI3K/Akt), mitogen-activated protein kinase/extracellular signal-regulated kinase (MAPK/ERK), AMP-activated protein kinase (AMPK), and nuclear factor-κB (NF-κB), as well as nuclear factor erythroid 2-related factor 2 (Nrf2)-mediated antioxidant signaling, which collectively regulate cell survival, proliferation, inflammation, metabolic adaptation, and antioxidant defense [6,13]. Persistent activation or dysregulation of these signaling networks may contribute to pathological cell survival and altered responses to cellular stress. Recent studies have also highlighted the importance of metabolic reprogramming and cellular adaptation in skin diseases. Alterations in glycolytic activity, mitochondrial energy production, and energy-sensing pathways are increasingly recognized as integral components of disease-associated cellular responses [13]. These metabolic alterations are tightly interconnected with oxidative stress and inflammatory signaling, thereby supporting cellular survival under pathological conditions.

In this context, the ability of skin cells to adapt to oxidative and metabolic stress represents an important determinant of disease progression and therapeutic responsiveness. Increasing evidence suggests that mitochondrial–nuclear communication and stress-responsive signaling pathways may contribute to the coordination of adaptive cellular responses to oxidative and metabolic stress [14]. Recent advances have also highlighted mitochondrial quality-control mechanisms, including mitophagy and mitochondrial fission–fusion dynamics, as important determinants of mitochondrial integrity and cellular adaptation under stress conditions [15]. These adaptive mechanisms may contribute to resistance against environmental and therapeutic stressors in various skin diseases [4,13]. Therefore, understanding the integration of metabolic reprogramming with intracellular signaling networks may provide valuable insights into the development of novel therapeutic strategies.

Although current therapeutic approaches can manage certain skin diseases, many strategies primarily target specific molecular pathways and may show limited efficacy because of adaptive or acquired resistance [4,5]. Unlike conventional pharmacological agents that are often designed to act on specific molecular targets, polyphenols can modulate multiple pathways associated with oxidative stress, mitochondrial function, inflammatory signaling, metabolic adaptation, and apoptosis-related responses [6,16]. These properties position polyphenols as promising candidates for modulating complex disease-associated cellular networks.

Accordingly, this review summarizes current mechanistic insights into the effects of polyphenols on oxidative stress, mitochondrial function, metabolic adaptation, and cell survival signaling in skin diseases. Particular attention is given to recent evidence concerning redox-sensitive signaling and mitochondrial quality-control mechanisms, while emphasizing findings from primary experimental studies where available. In addition, the therapeutic implications and current limitations of polyphenol-based approaches are discussed, with particular emphasis on the translational gap between experimental findings and physiological relevance.

## 2. Literature Search Strategy

A literature search was conducted to identify relevant studies addressing the effects of polyphenols on oxidative stress, mitochondrial function, cellular signaling, and skin disease pathophysiology. PubMed was used as the principal electronic database, supplemented by screening the reference lists of selected articles. Search terms included combinations of “polyphenols,” “flavonoids,” “resveratrol,” “quercetin,” “curcumin,” “epigallocatechin gallate,” “skin disease,” “skin cancer,” “melanoma,” “psoriasis,” “atopic dermatitis,” “photoaging,” “chronic wounds,” “oxidative stress,” “reactive oxygen species,” “mitochondrial dysfunction,” “mitophagy,” “mitochondrial dynamics,” “Nrf2,” “NF-κB,” “PI3K/Akt,” “MAPK/ERK,” and “AMPK.”

Studies were selected for inclusion when they provided mechanistic, experimental, or clinically relevant evidence concerning polyphenol-mediated regulation of oxidative stress, mitochondrial function, cellular signaling, or therapeutic responses in skin-related disease models. Primary experimental studies, including in vitro, in vivo, and clinical investigations, were prioritized for mechanistic and therapeutic conclusions, whereas review articles were used primarily to provide broader conceptual background and to identify additional relevant literature. Publications lacking direct relevance to the major mechanistic or disease-related themes of this review were not included.

Recent publications were preferentially considered to reflect current advances in the field, while selected earlier studies were retained when they provided foundational or directly relevant mechanistic evidence. The retrieved literature was evaluated according to its relevance to the major themes of this review, including oxidative stress and antioxidant defense, inflammatory and cell-survival signaling, mitochondrial metabolism and quality control, and the therapeutic and translational implications of polyphenols in skin diseases.

## 3. Molecular Pathophysiology of Skin Diseases

Skin diseases are characterized by complex and interconnected molecular mechanisms involving oxidative stress, inflammatory signaling, mitochondrial dysfunction, and dysregulated cellular pathways. Although these conditions differ in etiology and clinical manifestation, they share common molecular features that contribute to disease initiation, progression, and adaptive cellular responses under pathological stress conditions [2,3,4,5,6,9,12]. Understanding these molecular mechanisms provides an important framework for identifying potential therapeutic targets and evaluating multi-target therapeutic strategies.

### 3.1. Oxidative Stress and Redox Imbalance

Oxidative stress is a central component of the pathophysiology of skin diseases and arises from an imbalance between reactive oxygen species (ROS) production and antioxidant defense systems [6,11,17]. Environmental stressors, particularly ultraviolet (UV) radiation, are major sources of ROS generation in skin cells and induce damage to DNA, proteins, and lipids [6,7,17]. In addition, excessive ROS activates redox-sensitive signaling pathways involved in inflammation and cell survival [17,18].

Persistent oxidative stress contributes to chronic inflammatory responses and cellular dysfunction through activation of redox-sensitive transcription factors and signaling mediators [6,9,17,18]. Oxidative stress is also closely associated with mitochondrial dysfunction, thereby increasing ROS production and establishing a feedback loop that exacerbates cellular damage and disease progression [6,12,19].

Oxidative stress plays a critical role in activating signaling pathways associated with disease progression. Redox-sensitive pathways, including NF-κB and MAPK pathways, respond to increased intracellular ROS levels by regulating genes involved in inflammation, cell survival, and stress adaptation [6,17,18]. In parallel, nuclear factor erythroid 2-related factor 2 (Nrf2) functions as a major regulator of the cellular antioxidant response. Under oxidative stress, Nrf2 activation promotes its nuclear translocation and induces the expression of antioxidant and cytoprotective genes through the antioxidant response element (ARE), thereby enhancing cellular defense against oxidative damage [20,21]. This Nrf2-mediated antioxidant response supports major cellular defense systems, including superoxide dismutase (SOD), catalase (CAT), and glutathione (GSH)-related antioxidant capacity. Importantly, Nrf2 signaling interacts with inflammatory pathways, particularly NF-κB, and the crosstalk between these pathways contributes to the coordinated regulation of redox homeostasis and inflammatory responses in the skin [6,21]. Dysregulation of these signaling networks has been implicated in various skin disorders, including psoriasis, atopic dermatitis, and skin cancer [2,3,4,5,6,17,21].

These signaling events ultimately influence cellular outcomes such as inflammation, proliferation, and apoptosis, thereby contributing to disease progression and persistence.

### 3.2. Inflammatory Signaling Pathways

Chronic inflammation is a hallmark of many skin diseases and is driven by persistent activation of inflammatory signaling pathways [2,3,6,9,17]. A key regulator is nuclear factor-κB (NF-κB), which controls the expression of pro-inflammatory cytokines, chemokines, and other inflammatory mediators [6,18,21]. Activation of these signaling pathways promotes immune cell recruitment, sustained inflammatory responses, and tissue damage.

ROS also functions as an important signaling mediator that activates transcription factors and stress-responsive kinases [17,18]. In addition to NF-κB, MAPK signaling pathways, including p38 and c-Jun N-terminal kinase (JNK), contribute to inflammatory responses and cellular adaptation to environmental stressors [17,18]. Dysregulation of these interconnected signaling networks has been implicated in various skin disorders, including psoriasis and atopic dermatitis, in which persistent cytokine production and immune activation promote disease progression [2,3,21].

Collectively, these inflammatory signaling pathways regulate immune cell recruitment, cytokine amplification, and tissue remodeling, thereby contributing to chronic inflammation and impaired skin homeostasis.

### 3.3. Mitochondrial Dysfunction and Metabolic Stress

Mitochondria play essential roles in cellular energy production, redox regulation, and apoptosis [12,19,22]. In skin diseases, mitochondrial dysfunction is associated with impaired oxidative phosphorylation, excessive ROS production, and altered metabolic activity, thereby disrupting cellular energy balance and promoting oxidative damage [12,13,19,22].

Metabolic stress resulting from mitochondrial dysfunction further affects intracellular signaling and adaptive cellular responses [13,19]. Alterations in energy-sensing mechanisms, including AMP-activated protein kinase (AMPK), influence metabolic homeostasis and cell survival under stress conditions [6,13,19]. In addition to metabolic regulation, mitochondrial dysfunction is closely associated with activation of apoptosis-related signaling pathways. Disruption of mitochondrial integrity can activate mitochondria-dependent apoptotic signaling, involving pro-apoptotic factors and downstream caspase activation [19,23]. These molecular events ultimately contribute to apoptosis in damaged or stressed cells.

Beyond energy metabolism and apoptosis, mitochondrial quality-control mechanisms are increasingly recognized as critical determinants of cellular homeostasis under oxidative and metabolic stress. Mitochondrial dynamics, involving coordinated fission and fusion processes, contribute to the maintenance of mitochondrial integrity by facilitating the segregation of damaged mitochondrial components and the restoration of functional mitochondrial networks [15,19]. In parallel, selective removal of dysfunctional mitochondria through mitophagy, particularly through PTEN-induced kinase 1(PINK1)/Parkin-mediated pathways, limits the accumulation of damaged mitochondria and excessive ROS production [15,23]. Dysregulation of these processes may promote mitochondrial dysfunction, oxidative stress, and impaired cellular adaptation, thereby contributing to skin aging and disease pathogenesis [15,19,23]. Importantly, experimental evidence in human keratinocytes has demonstrated that impaired PINK1/Parkin-mediated mitophagy following UVB exposure leads to the accumulation of dysfunctional mitochondria, mtDNA leakage, and activation of downstream stress and apoptotic signaling [23].

Mitochondrial dysfunction is also integrated into intracellular signaling networks, including PI3K/Akt, MAPK, and AMPK pathways, which regulate cell survival, metabolism, and stress adaptation [6,13,19,24]. Dysregulation of these interconnected pathways has been implicated in skin aging, melanoma, and chronic inflammatory skin conditions [2,3,4,5,6,12,19]. These interactions highlight the central role of mitochondria in coordinating metabolic regulation, intracellular signaling, and cellular fate decisions under pathological conditions.

### 3.4. Cell Survival and Apoptotic Signaling

Cell survival and apoptosis are tightly regulated processes controlled by intracellular signaling pathways that integrate environmental and cellular stress signals [6,13,23,24,25]. PI3K/Akt and MAPK/ERK pathways play critical roles in regulating cell survival, proliferation, and stress adaptation [6,13,24]. Dysregulation of these pathways in skin diseases can contribute to abnormal cell survival, resistance to apoptosis, and uncontrolled cell growth [4,5,6,24].

Conversely, activation of apoptosis-related pathways, including mitochondria-dependent apoptotic mechanisms, promotes the elimination of damaged cells [23,25]. Apoptotic signaling is regulated by key molecular components, including Bcl-2 family proteins and downstream caspases, which collectively determine cellular fate under stress conditions [25]. Imbalance between pro-survival and pro-apoptotic signaling may result in pathological cell survival or excessive cell death, both of which are associated with disease development [6,23,25]. Dysregulation of these pathways has been reported in various skin diseases, including melanoma and chronic inflammatory disorders [2,3,4,5,6].

Furthermore, crosstalk among PI3K/Akt, MAPK/ERK, AMPK, redox-sensitive, and apoptosis-related pathways highlights the importance of signaling integration in maintaining cellular homeostasis [6,13,19,24]. These signaling pathways ultimately determine cellular outcomes, including proliferation, survival, and apoptosis, thereby contributing to disease progression and therapeutic responsiveness.

## 4. Polyphenols as Multi-Target Regulators of Cellular Signaling

### 4.1. General Properties of Polyphenols

Polyphenols are a structurally diverse group of bioactive compounds widely distributed in plant-derived foods, including fruits, vegetables, tea, and medicinal herbs [6,16,26]. In addition to terrestrial plant sources, polyphenolic compounds have also been identified in algae and fungi, which represent additional sources of structurally diverse bioactive phenolic compounds with antioxidant and anti-inflammatory activities [27,28]. These naturally occurring compounds contain one or more phenolic hydroxyl groups and aromatic rings, enabling interactions with diverse molecular targets involved in redox regulation and intracellular signaling [6,16,26].

Unlike conventional pharmacological agents that are often designed to act on specific molecular targets, polyphenols can modulate multiple interconnected signaling pathways. This characteristic is particularly relevant in complex diseases such as skin disorders, in which oxidative stress, inflammation, mitochondrial dysfunction, and dysregulated signaling pathways coexist. By targeting stress-responsive signaling pathways, polyphenols influence multiple cellular processes, including proliferation, survival, apoptosis, and metabolic adaptation [6,16].

Representative polyphenols, including resveratrol, quercetin, curcumin, epigallocatechin gallate (EGCG), genistein, apigenin, and luteolin, have been reported to regulate oxidative stress, inflammatory signaling, and mitochondrial function in diverse experimental models [6,16,26].

**Figure 1:**
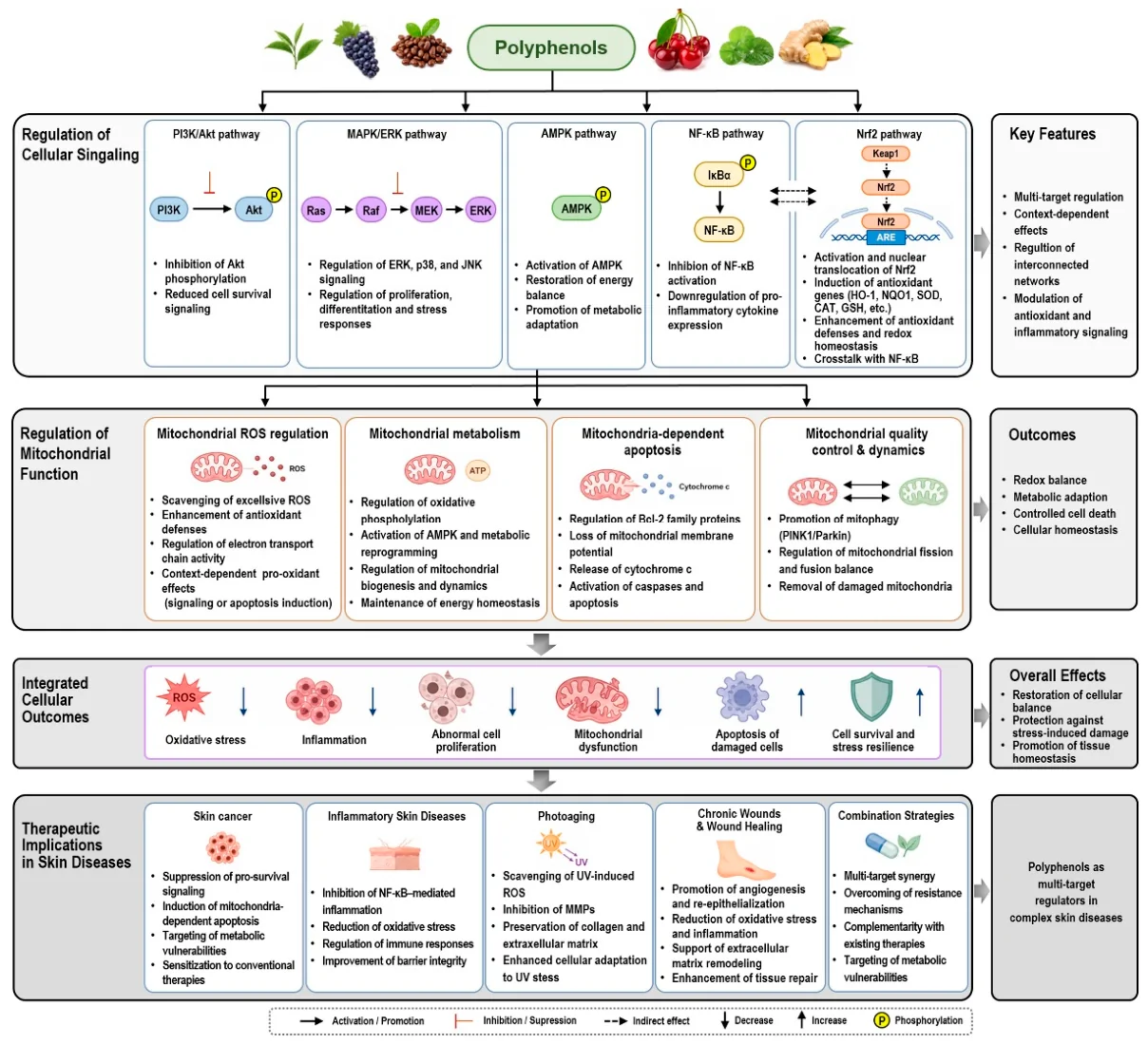
Schematic overview of polyphenol-mediated regulation of cellular signaling and mitochondrial function in skin diseases. Polyphenols influence cellular signaling pathways, including PI3K/Akt, MAPK/ERK, AMPK, NF-κB, and Nrf2-mediated antioxidant signaling, and regulate mitochondrial function through modulation of mitochondrial ROS, metabolic activity, mitochondrial dynamics, and apoptosis-related signaling pathways, thereby contributing to redox homeostasis and cellular adaptation under stress conditions. Activation of Nrf2-mediated antioxidant defenses contributes to the maintenance of redox homeostasis and interacts with inflammatory signaling, including NF-κB. These effects are associated with reduced oxidative stress, inflammatory responses, abnormal cell proliferation, and mitochondrial dysfunction, while promoting apoptosis in damaged or malignant cells. Collectively, these regulatory mechanisms may contribute to therapeutic responses in skin cancer, inflammatory skin diseases, photoaging, and chronic wound healing. Arrows indicate activation or promotion, whereas blunt-ended lines indicate inhibition or suppression. Created in BioRender. Lee, Y. J. (2026) https://BioRender.com/q56928w.

Figure 1 illustrates the integrated effects of polyphenols on cellular signaling pathways and mitochondrial function involved in the pathogenesis of skin diseases. Through regulation of oxidative stress, inflammatory signaling, metabolic activity, and apoptosis-related pathways, polyphenols influence cellular outcomes such as inflammation, proliferation, survival, and apoptosis. In particular, activation of the Nrf2-mediated antioxidant response represents an important mechanism through which polyphenols enhance endogenous antioxidant defenses and modulate redox-sensitive inflammatory signaling [6,16].

Polyphenols encompass several subclasses, including flavonoids, phenolic acids, stilbenes, and lignans, which possess distinct chemical structures and biological activities [16,26]. One of the defining characteristics of polyphenols is their ability to regulate redox homeostasis. Although traditionally recognized as antioxidants, polyphenols may also exert pro-oxidant effects under specific conditions, particularly at high concentrations or in the presence of transition metals [6,16]. Depending on the cellular environment and redox status, these effects may protect cells from oxidative damage or promote apoptosis in damaged or malignant cells.

In addition to redox regulation, polyphenols can interact with kinases, transcription factors, metabolic enzymes, and other signaling proteins [6,16]. These interactions enable polyphenols to influence interconnected signaling pathways and complex cellular responses. Importantly, the biological effects of polyphenols are strongly affected by factors such as concentration, bioavailability, metabolism, and cellular microenvironment, highlighting the need for careful interpretation of experimental findings and their translational relevance [16].

### 4.2. Regulation of PI3K/Akt and MAPK/ERK Pathways

The PI3K/Akt and MAPK/ERK signaling pathways play central roles in regulating cell survival, proliferation, and stress responses. Dysregulation of these pathways is commonly observed in skin diseases and contributes to abnormal cell growth, resistance to apoptosis, and sustained inflammatory responses [4,6,24].

Polyphenols influence the PI3K/Akt pathway through regulation of upstream receptor signaling and downstream effectors involved in cell survival and metabolism. Recent evidence further indicates that polyphenol-mediated regulation of PI3K/Akt signaling is functionally interconnected with downstream mTOR and FOXO signaling and with SIRT1-dependent stress-response mechanisms. These interactions may influence cellular metabolism, antioxidant defense, autophagy, and survival under oxidative and metabolic stress conditions [6,16,24]. In experimental models, several polyphenols have been shown to modulate Akt phosphorylation and related pro-survival signaling, with the direction and magnitude of these effects depending on the compound, dose, and cellular context [6,29,30,31]. Recent experimental evidence in cutaneous melanoma further supports the ability of flavonoids such as quercetin and luteolin to modulate PI3K/Akt- and ERK-related signaling [29,30,31].

Polyphenols also regulate the MAPK/ERK pathway, which is closely associated with cell proliferation, differentiation, and stress responses. Depending on cellular context and exposure conditions, modulation of ERK signaling by polyphenols may influence cell proliferation and apoptosis [6,24,29]. In addition to ERK, polyphenols may influence other MAPK family members, including p38 and c-Jun N-terminal kinase (JNK), thereby affecting stress-responsive signaling pathways [6,24].

**Figure 2:**
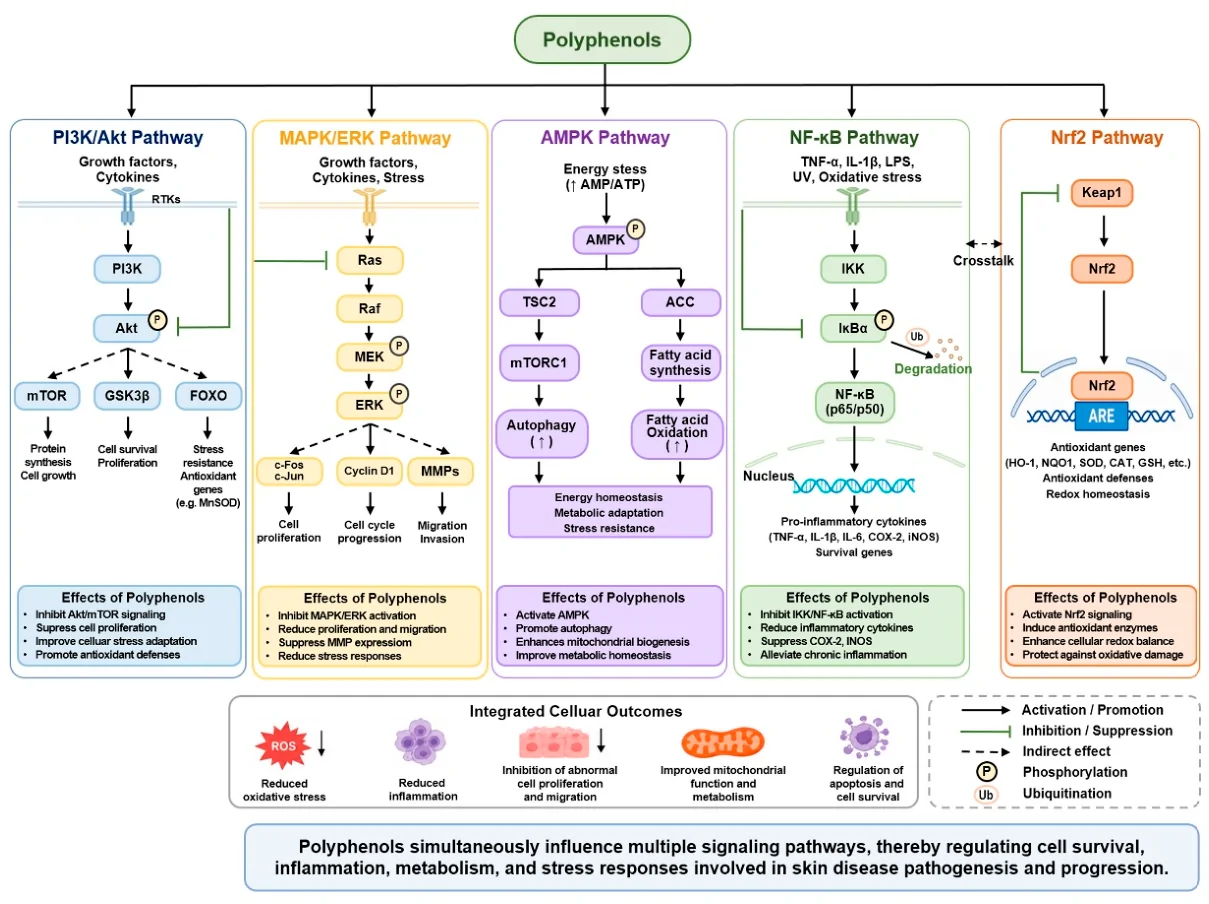
Polyphenol-mediated regulation of major cellular signaling pathways involved in skin disease pathogenesis. Polyphenols modulate Akt phosphorylation and downstream signaling in the PI3K/Akt pathway, thereby influencing pro-survival and proliferative signaling. In the MAPK/ERK pathway, polyphenols regulate kinase activity and downstream transcriptional targets associated with cell proliferation, migration, and stress adaptation. Polyphenols also activate AMPK, a key regulator of cellular energy homeostasis, thereby promoting metabolic adaptation and stress responses under energy-deficient conditions. In addition, inhibition of NF-κB signaling by polyphenols reduces the expression of pro-inflammatory cytokines and inflammatory mediators associated with chronic inflammation. Polyphenols can also activate Nrf2-mediated antioxidant signaling, promoting the expression of antioxidant and cytoprotective genes and contributing to redox homeostasis. Crosstalk between Nrf2 and NF-κB further links antioxidant defense with the regulation of inflammatory responses. Through effects on these interconnected signaling pathways, polyphenols may reduce oxidative stress, inflammation, abnormal cell proliferation, and metabolic dysregulation associated with skin diseases. Arrows indicate activation or promotion, whereas blunt-ended lines indicate inhibition or suppression. Created in BioRender. Lee, Y. J. (2026) https://BioRender.com/kwc74sa.

Figure 2 summarizes the effects of polyphenols on interconnected signaling pathways, including PI3K/Akt, MAPK/ERK, AMPK, NF-κB, and Nrf2-mediated antioxidant signaling. Through regulation of these signaling systems, polyphenols influence cellular processes such as survival, proliferation, inflammation, metabolic adaptation, and antioxidant defense. These findings support the role of polyphenols as multi-target regulators of complex intracellular signaling networks rather than simple inhibitors or activators of individual pathways.

In addition to direct regulation of Akt and ERK signaling, polyphenols may influence upstream receptor-mediated signaling and downstream transcriptional responses, thereby affecting cellular adaptation to oxidative and metabolic stress. Integration of these pathways with redox signaling and metabolic regulation further highlights the complex effects of polyphenols in skin diseases [6,16,24,29,30,31].

Emerging evidence also suggests that polyphenols influence downstream signaling components such as mechanistic target of rapamycin (mTOR) and stress-activated kinases including p38 and JNK [6,24]. These signaling pathways are closely associated with oxidative stress responses, metabolic adaptation, and cell survival, supporting the role of polyphenols as multi-target regulators of intracellular signaling pathways.

### 4.3. AMPK and Metabolic Stress Signaling

AMP-activated protein kinase (AMPK) is a key regulator of cellular energy homeostasis and plays an essential role in metabolic adaptation to stress. Activation of AMPK promotes catabolic processes while suppressing anabolic pathways, thereby contributing to the restoration of cellular energy balance in response to metabolic stress [6,13].

Polyphenols can influence AMPK activity either directly or indirectly through alterations in cellular energy status and redox balance [6,16]. Activation of AMPK by certain polyphenols can modulate cell growth, metabolic adaptation, and cellular responses to stress. In skin pathophysiology, AMPK signaling is particularly relevant because of its involvement in the regulation of cellular metabolism, oxidative stress, inflammation, and survival [6,13].

Representative polyphenols such as resveratrol have been reported to activate AMPK-related signaling and support cellular adaptation under oxidative stress conditions [32,33]. In primary human keratinocytes, resveratrol prevented oxidative stress-induced senescence and proliferative dysfunction through an AMPK–FOXO3-associated mechanism, providing direct experimental evidence for this pathway in skin cells [32].

Moreover, AMPK signaling interacts with multiple intracellular pathways, including PI3K/Akt, MAPK, and mTOR-related signaling, thereby coordinating metabolic and stress-responsive responses [6,13,24]. Through regulation of AMPK and its downstream targets, polyphenols may influence cellular adaptation to metabolic stress and disease-associated metabolic alterations.

### 4.4. NF-κB and Inflammatory Signaling

Nuclear factor-κB (NF-κB) is a central regulator of inflammatory signaling and plays a pivotal role in the pathogenesis of many skin diseases. Activation of NF-κB promotes transcription of pro-inflammatory cytokines, chemokines, and other inflammatory mediators, thereby contributing to inflammatory responses and immune-cell recruitment [6,18,21].

Polyphenols can suppress NF-κB activation through regulation of upstream kinases and signaling intermediates involved in inflammatory pathways. Inhibition of NF-κB signaling by polyphenols can reduce the expression of inflammatory mediators and attenuate inflammatory responses [6,16]. Representative polyphenols, including luteolin and curcumin, have been reported to modulate NF-κB-mediated inflammatory signaling [6,16,34,35]. Recent experimental evidence in a psoriasis-like HaCaT model demonstrated that luteolin suppressed inflammatory responses through regulation of the TRAF6/TAK1/IKK/IκB–NF-κB signaling axis and improved the expression of skin-barrier-associated proteins [34].

NF-κB signaling is closely interconnected with oxidative stress and other intracellular signaling pathways, including PI3K/Akt, MAPK, and Nrf2 signaling [6,18,21]. Through modulation of NF-κB activity and its crosstalk with redox-sensitive pathways, polyphenols may influence inflammatory responses, oxidative stress, and cell-survival signaling. Dysregulated NF-κB signaling has been strongly implicated in chronic inflammatory skin diseases, in which sustained cytokine production contributes to disease persistence and progression [2,3,6,18,21].

## 5. Polyphenol-Mediated Regulation of Mitochondrial Function

Mitochondria play a central role in cellular homeostasis by regulating energy production, redox balance, and apoptosis. In skin diseases, mitochondrial dysfunction is closely associated with increased oxidative stress, disrupted metabolic activity, and impaired cellular stress adaptation [12,19,22].

Polyphenols influence mitochondrial function through multiple interconnected mechanisms, including regulation of excessive mitochondrial ROS production, metabolic pathways, and apoptosis-related signaling [6,16]. Emerging evidence further indicates that polyphenols can influence mitochondrial quality-control mechanisms, including mitophagy and mitochondrial dynamics, which are essential for maintaining mitochondrial integrity under cellular stress [15,36]. These effects collectively influence cellular responses to oxidative and metabolic stress [6,15,16,36]. Representative polyphenols, including resveratrol, quercetin, curcumin, and epigallocatechin gallate (EGCG), have been reported to regulate redox homeostasis, metabolic activity, and apoptosis-associated signaling pathways [6,16,36].

**Figure 3:**
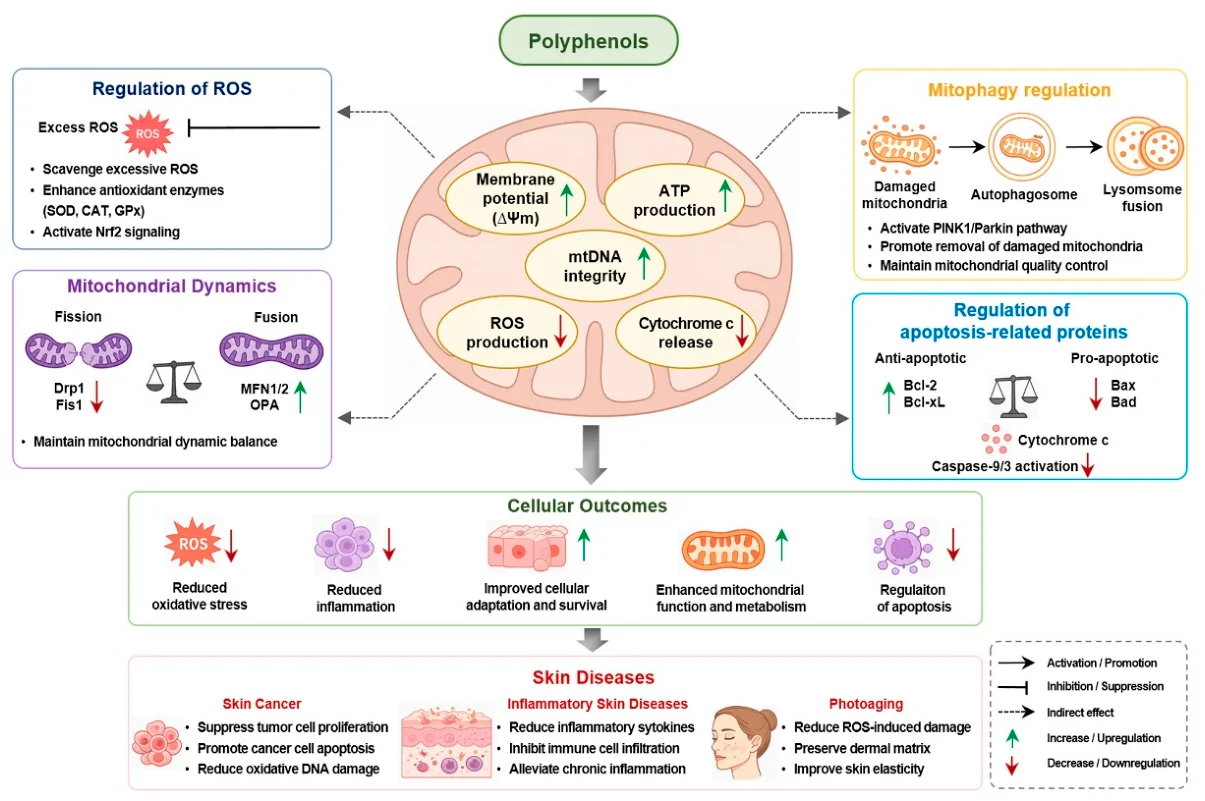
Polyphenol-mediated effects on mitochondrial function and cellular responses in skin diseases. Polyphenols reduce excessive ROS production and enhance antioxidant defense systems, thereby maintaining intracellular redox homeostasis. In addition, polyphenols influence mitochondrial membrane potential, ATP production, and protection of mitochondrial DNA (mtDNA), thereby supporting mitochondrial integrity and metabolic adaptation under stress conditions. Polyphenols also support mitochondrial quality control through regulation of mitochondrial fission–fusion balance and mitophagy-related pathways, including the PINK1/Parkin pathway. Furthermore, polyphenols regulate mitochondria-dependent apoptosis through modulation of cytochrome c release, Bcl-2 family proteins, and downstream caspase activation. These effects are associated with reduced oxidative stress and inflammation, improved mitochondrial function, enhanced cell survival, and apoptosis of damaged cells, which collectively contribute to protective responses in skin diseases. Arrows indicate activation or promotion, whereas blunt-ended lines indicate inhibition or suppression. Created in BioRender. Lee, Y. J. (2026) https://BioRender.com/k48a2nf.

These mitochondrial regulatory mechanisms are particularly relevant in skin diseases, where oxidative stress, metabolic dysregulation, and inflammatory signaling contribute to disease progression [6,12,19,22]. Figure 3 summarizes the effects of polyphenols on mitochondrial ROS regulation, metabolic adaptation, mitochondrial quality control, and apoptosis-related signaling pathways involved in cellular responses to oxidative and metabolic stress.

### 5.1. Mitochondrial ROS Regulation

Mitochondria are an important source of intracellular ROS, which can be generated during oxidative phosphorylation. Under pathological conditions, excessive mitochondrial ROS production contributes to oxidative damage, activation of inflammatory signaling pathways, and cellular dysfunction [6,12,19].

Polyphenols influence mitochondrial ROS levels through multiple mechanisms. As redox-active compounds, polyphenols can modulate ROS levels and enhance endogenous antioxidant defense systems, thereby reducing oxidative damage [6,16]. In addition, polyphenols may limit excessive ROS generation by preserving mitochondrial function and modulating processes associated with mitochondrial electron transport and redox homeostasis [6,16,36,37,38,39]. Representative polyphenols, including quercetin, resveratrol, and EGCG, have been reported to modulate mitochondrial or intracellular ROS production and redox balance in experimental models [37,38,39,40,41,42].

Interestingly, under certain conditions, polyphenols may also induce moderate increases in ROS levels that function as signaling mediators involved in adaptive stress responses or promote apoptosis in damaged or malignant cells [6,16,42]. These findings highlight the context-dependent role of ROS as both a mediator of cellular damage and an intracellular signaling molecule.

### 5.2. Mitochondrial Metabolism

Mitochondria are essential regulators of cellular energy metabolism, primarily through oxidative phosphorylation (OXPHOS). In skin diseases, mitochondrial metabolic function can be disrupted, resulting in dysregulated energy production and impaired metabolic flexibility [12,13,19,22].

Polyphenols influence mitochondrial metabolism through regulation of metabolic pathways and energy-sensing mechanisms. Through interactions with signaling pathways such as AMPK, polyphenols may promote metabolic adaptation and support cellular energy homeostasis under stress conditions [6,32,33,41]. Depending on the cellular context, polyphenols may enhance mitochondrial function in non-malignant cells or suppress metabolic pathways supporting the survival of transformed cells [6,13,16].

Experimental evidence indicates that polyphenols can modulate glycolysis and mitochondrial metabolism in malignant cells, thereby influencing adenosine triphosphate (ATP) production and cell-death signaling [6,13,16]. In addition, polyphenols may influence mitochondrial biogenesis and mitochondrial dynamics, including fusion and fission processes required for maintenance of mitochondrial integrity and function [15,19,36].

These findings suggest that polyphenols influence mitochondrial metabolism in a context-dependent manner. Such metabolic adaptations are closely associated with cellular stress responses and highlight the role of mitochondrial metabolism in coordinating energy balance and adaptive signaling under pathological conditions [6,13,19].

Beyond metabolic regulation, mitochondrial quality control is essential for preserving a functional mitochondrial network under oxidative and metabolic stress. Selective removal of damaged mitochondria through mitophagy, particularly through the PINK1/Parkin pathway, limits the accumulation of dysfunctional mitochondria and excessive ROS [15,23]. Mitochondrial fission and fusion further maintain mitochondrial homeostasis by facilitating the segregation of damaged mitochondrial components and restoration of functional mitochondrial networks [15,19]. Recent evidence suggests that polyphenols may modulate these quality-control processes by influencing mitophagy and mitochondrial dynamics under stress conditions [36]. These mechanisms provide an additional link between polyphenol-mediated mitochondrial regulation and cellular adaptation in skin pathophysiology.

### 5.3. Mitochondria-Dependent Apoptosis

Mitochondria play a pivotal role in the regulation of intrinsic apoptosis through control of mitochondrial membrane integrity and release of pro-apoptotic factors. Disruption of mitochondrial membrane potential can promote cytochrome c release and activation of downstream caspase cascades, ultimately leading to apoptosis [19,25].

Polyphenols influence mitochondria-dependent apoptotic pathways through regulation of key apoptotic proteins, including Bcl-2 family proteins and downstream caspases [6,16,25]. In many experimental models, polyphenols promote apoptosis in damaged or malignant cells by disrupting mitochondrial homeostasis and activating apoptosis-related signaling pathways [6,16,42,43,44,45]. Conversely, in non-malignant cells exposed to oxidative stress, some polyphenols may preserve mitochondrial function and limit excessive cell death, emphasizing the context-dependent nature of their biological effects [6,16,37,38,41,42,46,47,48].

Representative polyphenols, including curcumin and quercetin, have been reported to affect oxidative-stress- and apoptosis-related signaling and cellular stress responses [6,16,29,30,39,49]. These findings highlight the role of polyphenols in regulating cellular fate under oxidative and metabolic stress conditions. Through effects on mitochondrial integrity and apoptosis-related signaling pathways, polyphenols may influence cellular adaptation, survival, and programmed cell death.

A summary of representative polyphenols and their effects on cellular signaling pathways and mitochondrial function is presented in Table 1.

## 6. Therapeutic Implications in Skin Diseases

The multi-target regulatory properties of polyphenols provide a mechanistic basis for their potential therapeutic applications in skin diseases. Through coordinated effects on oxidative stress, mitochondrial function, inflammatory signaling, metabolic adaptation, and cell-death pathways, polyphenols may influence multiple pathogenic mechanisms associated with disease progression and cellular stress responses [6,12,13,16,36]. These integrated regulatory effects are particularly relevant in skin cancer, inflammatory skin diseases, photoaging, and chronic wounds, where oxidative stress, dysregulated signaling pathways, mitochondrial dysfunction, and metabolic imbalance contribute to pathological progression [2,3,4,5,6,12,13]. The following sections summarize current evidence regarding the therapeutic implications of polyphenols in major skin disease contexts.

### 6.1. Skin Cancer

Skin cancers, including melanoma and non-melanoma skin cancers, are characterized by dysregulated cell proliferation, resistance to apoptosis, and metabolic reprogramming that supports tumor growth and survival. Aberrant activation of signaling pathways such as PI3K/Akt and MAPK/ERK contributes to enhanced cell survival and proliferation, while mitochondrial alterations promote oxidative stress tolerance and metabolic flexibility [4,5,6,13,24]. These mechanisms are particularly relevant in melanoma, where tumor cells exhibit substantial metabolic adaptability and may develop resistance to oxidative and therapeutic stress [4,13].

Polyphenols have been reported to interfere with these oncogenic processes through modulation of pro-survival signaling, redox homeostasis, and mitochondrial function [6,16]. Representative compounds, including quercetin, luteolin, resveratrol, curcumin, and epigallocatechin gallate (EGCG), have demonstrated antiproliferative or pro-apoptotic effects through regulation of PI3K/Akt, MAPK/ERK, NF-κB, and apoptosis-related pathways [6,16,29,30,31,43,44,45,52]. Recent experimental evidence in A375 human cutaneous melanoma cells further demonstrated that quercetin and luteolin modulate ERK- and PI3K/Akt-associated signaling, supporting the context-dependent effects of flavonoids on melanoma cell survival and proliferation [29].

In addition, polyphenols can influence mitochondria-dependent apoptosis through alterations in mitochondrial function, Bcl-2 family proteins, and downstream caspase signaling [6,16,25,42,43,44,45]. Their ability to regulate cellular redox balance is also important because cancer cells frequently rely on adaptive antioxidant mechanisms to survive under conditions of elevated oxidative stress. Depending on concentration and cellular context, certain polyphenols may exert pro-oxidant effects that overwhelm the antioxidant capacity of malignant cells and thereby promote cell death [6,16,43,44].

Collectively, these findings suggest that polyphenols may target multiple vulnerabilities in skin cancer cells, including signaling dysregulation, metabolic adaptation, mitochondrial dysfunction, and oxidative stress resistance. However, much of the available mechanistic evidence remains derived from in vitro and preclinical studies, and additional in vivo and clinical investigations are required to establish effective doses, pharmacokinetic properties, and therapeutic relevance in patients [4,16].

### 6.2. Inflammatory Skin Diseases

Inflammatory skin diseases, including psoriasis and atopic dermatitis, are characterized by persistent immune activation, oxidative stress, barrier dysfunction, and dysregulated inflammatory signaling [2,3,6,21]. In psoriasis, cytokine networks involving TNF-α, IL-17, and related inflammatory mediators contribute to sustained keratinocyte activation and immune-cell recruitment, whereas atopic dermatitis involves complex interactions among epidermal barrier impairment, immune dysregulation, and environmental stress [2,3].

NF-κB represents an important molecular link between oxidative stress and inflammatory signaling in these disorders. Persistent NF-κB activation promotes the expression of pro-inflammatory cytokines, chemokines, and other inflammatory mediators, while its crosstalk with Nrf2-dependent antioxidant signaling contributes to the regulation of the balance between inflammation and cellular redox defense [6,21].

Polyphenols may disrupt this oxidative stress–inflammation feedback loop by reducing excessive ROS accumulation, modulating inflammatory signaling, and enhancing endogenous antioxidant responses [6,16,21]. Representative polyphenols and flavonoids have demonstrated anti-inflammatory effects in experimental skin-disease models [6,16,34,53]. Recent experimental evidence in psoriasis-like keratinocyte models further supports the ability of polyphenolic compounds to regulate inflammatory signaling and abnormal keratinocyte responses [34,53]. In particular, rutin suppressed M5-induced inflammatory mediator expression and abnormal differentiation in HaCaT keratinocytes through regulation of JAK2/STAT3 signaling [53].

In addition to suppressing inflammatory signaling, polyphenols may influence epidermal barrier function and innate and adaptive immune responses. Such multi-target actions are particularly relevant to inflammatory skin disorders in which oxidative stress, inflammatory signaling, and barrier dysfunction are closely interconnected [2,3,6,16,34].

Despite these promising findings, substantial differences in compound bioavailability, metabolism, formulation, dose, and experimental models limit direct translation to clinical practice. Further well-designed animal and clinical studies are therefore required to establish the therapeutic efficacy and safety of individual polyphenols in psoriasis, atopic dermatitis, and other chronic inflammatory skin diseases [16].

### 6.3. Photoaging

Photoaging is primarily induced by chronic exposure to ultraviolet (UV) radiation and is characterized by excessive ROS generation, DNA damage, mitochondrial dysfunction, inflammation, and degradation of extracellular matrix components [6,7,15,19]. UV-induced activation of redox-sensitive signaling pathways, including MAPK and NF-κB, promotes matrix metalloproteinase (MMP) expression and contributes to collagen degradation and structural alterations in the skin [6,17,18].

Polyphenols may protect against photoaging through multiple complementary mechanisms, including attenuation of oxidative stress, enhancement of antioxidant defenses, suppression of inflammatory signaling, and preservation of extracellular matrix integrity [6,16,38,40,41,42,46,48,51]. Recent primary studies provide direct experimental evidence for these effects in skin-related models. EGCG reduced UV-induced oxidative damage in human skin fibroblasts and related experimental models [38,48], whereas resveratrol reduced ROS production and collagen degradation and promoted AMPK-dependent autophagy in UVA-exposed human skin fibroblasts and mouse skin [41].

Beyond antioxidant and anti-inflammatory effects, recent evidence suggests that polyphenols may also modulate cellular responses to UV-induced DNA damage. In particular, polyphenol-mediated regulation of DNA damage-response mechanisms, including histone H2A variant X (H2AX) phosphorylation, may facilitate recognition and repair of damaged DNA and thereby contribute to maintenance of genomic integrity under oxidative stress [46].

Mitochondrial protection may provide an additional mechanism underlying the photoprotective effects of polyphenols. Because UV-induced mitochondrial dysfunction can enhance ROS production and impair cellular stress adaptation, preservation of mitochondrial integrity, redox homeostasis, autophagy, and mitochondrial quality-control mechanisms may contribute to protection against chronic photodamage [15,19,23,36,41]. Notably, the recent resveratrol study provides direct evidence linking AMPK-dependent autophagy with reduced ROS production and protection against UVA-induced photoaging [41].

Nevertheless, the clinical application of polyphenols for photoaging remains limited by issues including bioavailability, chemical stability, skin penetration, formulation, and differences between experimental exposure conditions and physiologically achievable concentrations. Improved topical formulations and delivery technologies, together with controlled human studies, are therefore required to determine their clinical effectiveness [16,51].

### 6.4. Chronic Wounds and Wound Healing

Chronic wounds, including diabetic foot ulcers, are characterized by persistent inflammation, excessive oxidative stress, impaired angiogenesis, extracellular matrix dysregulation, and defective re-epithelialization, all of which contribute to delayed tissue repair and prolonged wound persistence [54,55]. In diabetic wounds, oxidative stress and inflammatory signaling can impair keratinocyte and fibroblast function and interfere with the coordinated cellular responses required for effective wound repair [54,55]. Nrf2 is particularly relevant because it regulates antioxidant defense, inflammatory responses, keratinocyte proliferation and migration, and angiogenesis during diabetes-associated wound healing [54].

The antioxidant and anti-inflammatory properties of polyphenols provide a mechanistic basis for their potential application in chronic wound management. Polyphenols may promote wound repair by reducing excessive oxidative and inflammatory stress while supporting cell proliferation, migration, angiogenesis, re-epithelialization, and extracellular matrix remodeling [54,55,56,57]. Regulation of redox-sensitive pathways, including Nrf2, and other wound-associated signaling mechanisms may further contribute to restoration of tissue homeostasis [54,55,56,57]. Recent primary evidence demonstrated that resveratrol improved proliferation and migration of high-glucose-exposed human dermal fibroblasts and accelerated diabetic wound healing through inhibition of Notch signaling [56]. Curcumin likewise promoted diabetic wound healing through a miR-152-3p/FBN1/TGF-β-associated mechanism in fibroblast and animal models [57].

Recent advances in biomaterial-based delivery systems have further expanded the potential application of polyphenols in wound care [55,58,59,60]. A resveratrol-loaded ionic liquid/GelMA hydrogel demonstrated controlled release, antibacterial activity, PI3K/Akt-associated angiogenic effects, reduced inflammation, and enhanced wound closure in a diabetic mouse model [58]. A biopolymeric scaffold containing the phenolic compound p-coumaric acid promoted fibroblast responses and diabetic wound healing while modulating MMP-9 and TGF-β3 expression [59]. In addition, a curcumin-containing multifunctional hydrogel promoted diabetic wound healing through antimicrobial, antioxidant, anti-inflammatory, and pro-angiogenic effects [60].

These biomaterial-based approaches are particularly relevant to diabetic and other chronic wounds, in which prolonged oxidative stress, inflammation, microbial burden, and impaired tissue regeneration limit normal healing. Nevertheless, further preclinical optimization and well-controlled clinical studies are required to establish formulation stability, dosing, biocompatibility, long-term safety, and therapeutic efficacy before these approaches can be translated into routine wound care [55,58,59,60].

### 6.5. Combination Strategies

Given the multifactorial nature of skin-disease pathophysiology, therapeutic strategies capable of simultaneously targeting multiple molecular and metabolic pathways are increasingly attractive. The multi-target properties of polyphenols make them potential candidates for combination approaches with conventional therapies, other bioactive compounds, or advanced drug-delivery systems [6,16,36,55].

In cancer and inflammatory disease models, polyphenols have been investigated for their potential to enhance apoptosis, suppress inflammatory signaling, and modify cellular responses to therapeutic stress [6,16,43,52]. Such approaches may be particularly relevant when adaptive antioxidant mechanisms, metabolic plasticity, and pro-survival signaling contribute to therapeutic resistance.

Targeting mitochondrial function and metabolic adaptation represents another potential strategy. Polyphenols can influence AMPK-related metabolic signaling, redox homeostasis, mitochondrial function, autophagy, and mitochondrial quality-control processes [15,32,36,41]. These interconnected effects suggest that polyphenols may complement therapeutic approaches directed toward metabolic or oxidative vulnerabilities in pathological cells.

Advances in nanoformulation, encapsulation, hydrogel, and other delivery technologies may further improve the stability, bioavailability, tissue targeting, and controlled release of polyphenols [52,55,58,59,60]. However, combination strategies require careful evaluation because interactions between polyphenols and conventional treatments may vary according to compound concentration, timing, pharmacokinetics, formulation, and disease context. Future studies should therefore prioritize mechanistically informed combinations supported by appropriate pharmacokinetic evaluation, in vivo models, and ultimately controlled clinical investigations [16,55].

A summary of the key mechanisms underlying polyphenol-mediated regulation of cellular signaling and mitochondrial function in skin diseases is presented in Table 2.

## 7. Limitations and Translational Considerations

Despite growing evidence supporting the effects of polyphenols on cellular signaling, oxidative stress, mitochondrial function, and metabolic regulation, several important limitations should be considered when evaluating their therapeutic potential in skin diseases. One major limitation is the discrepancy between experimental conditions and physiological relevance, particularly regarding the concentrations used in in vitro studies. Many reported biological effects are observed at micromolar concentrations that may be difficult to achieve in human tissues through dietary intake or conventional administration routes because of limited absorption, rapid metabolism, and systemic elimination [16,61,62,63,64,65].

Another critical issue is the limited bioavailability of many polyphenols. Following oral administration, these compounds may undergo extensive intestinal and hepatic metabolism, including conjugation and degradation, which can substantially alter the concentrations and molecular forms reaching target tissues [61,62,63,64,65]. For dermatological applications, limited chemical stability, formulation-dependent release, and insufficient penetration across the skin barrier represent additional challenges. These limitations have stimulated the development of topical formulations, encapsulation systems, nanoparticles, and other delivery approaches designed to improve compound stability and local bioavailability [16,52,55,58,59,60,66].

The dual antioxidant and pro-oxidant properties of polyphenols introduce additional complexity. Their biological effects can vary according to compound structure, concentration, exposure duration, cellular redox status, and disease context [6,16,61]. Whereas antioxidant activity may protect non-malignant cells from excessive oxidative stress, pro-oxidant effects under particular experimental conditions may contribute to the induction of cell death in malignant cells. Consequently, therapeutic responses observed for one polyphenol or experimental model should not necessarily be generalized to other compounds or disease settings [6,16].

Another important limitation is the predominance of in vitro and preclinical studies and the relative lack of robust clinical evidence supporting efficacy in skin diseases [16]. Experimental cell and animal models provide essential mechanistic information but do not fully reproduce the complexity of human skin physiology, pharmacokinetics, disease heterogeneity, or long-term therapeutic exposure. Accordingly, mechanistic conclusions should be interpreted primarily in the context of evidence from primary experimental studies, while findings derived from review articles should serve mainly to provide broader conceptual and methodological context. Well-designed in vivo studies and controlled clinical investigations are therefore necessary to determine whether experimentally observed effects can be achieved safely and effectively in humans [16,51,61,62,66].

From a translational perspective, development of effective delivery systems represents an important area for future investigation. Nanoformulations, encapsulation strategies, hydrogels, and other targeted or localized delivery platforms may improve stability, controlled release, tissue retention, and local bioavailability of polyphenols [52,55,58,59,60,66]. The recent development of polyphenol-containing wound dressings provides direct experimental evidence for the feasibility of localized delivery in skin-related applications [58,59,60]. Nevertheless, improvements in delivery efficiency should be accompanied by rigorous evaluation of formulation characteristics, biocompatibility, pharmacokinetics, tissue distribution, dose–response relationships, and long-term safety before clinical translation [55,61,62,66].

Finally, because polyphenols can influence multiple intracellular pathways, their interactions with conventional therapeutic agents require careful investigation. Combination strategies may potentially enhance therapeutic efficacy through complementary effects on oxidative stress, inflammatory signaling, mitochondrial function, and cell-survival pathways. However, such interactions may also be additive, synergistic, or antagonistic depending on the compounds, doses, timing, and disease context [16,43,52]. Therefore, mechanistically informed combination studies, supported by appropriate pharmacokinetic and safety evaluations, are required before polyphenol-based combination strategies can be translated into clinical practice.

Collectively, addressing these limitations will be essential for bridging the gap between mechanistic findings and clinical application. Future research should prioritize physiologically relevant experimental concentrations, standardized formulations, improved delivery technologies, rigorous pharmacokinetic characterization, and well-designed clinical studies to establish the safety and therapeutic efficacy of polyphenol-based interventions for skin diseases.

## 8. Conclusions

Polyphenols have emerged as multi-target regulators of cellular processes critically involved in the pathogenesis of skin diseases. Through effects on oxidative stress, mitochondrial function, inflammatory signaling, and cell survival pathways, these bioactive compounds influence key molecular networks associated with cellular responses to environmental and metabolic stressors. This multi-target mode of action may provide advantages in modulating complex disease-associated networks compared with therapeutic approaches directed toward specific molecular targets and highlights the potential relevance of polyphenols in complex skin disorders.

Accumulating evidence indicates that polyphenols affect interconnected signaling pathways, including PI3K/Akt, MAPK/ERK, AMPK, NF-κB, and Nrf2 pathways, thereby influencing proliferation, apoptosis, inflammation, metabolic adaptation, and antioxidant defense. Their effects on mitochondrial function and redox homeostasis further contribute to the maintenance of cellular integrity under pathological conditions. Emerging evidence also highlights mitochondrial quality-control processes, including mitophagy and mitochondrial dynamics, as additional mechanisms through which polyphenols may influence cellular adaptation to oxidative and metabolic stress. Collectively, these findings provide an important mechanistic framework for understanding how polyphenols influence multiple processes associated with skin diseases.

Despite these promising findings, several challenges remain regarding clinical translation. Limitations related to bioavailability, pharmacokinetics, and discrepancies between experimental and physiologically relevant conditions must be carefully addressed. In addition, variability in polyphenol activity according to compound, dose, cellular context, and metabolic conditions highlights the importance of defining appropriate conditions for therapeutic efficacy and safety. Future studies should therefore focus on bridging mechanistic findings with clinical application through well-designed in vivo investigations and controlled clinical studies. Furthermore, the development of advanced delivery systems and rational combination strategies may improve the therapeutic applicability of polyphenols in skin diseases.

In conclusion, polyphenols represent promising multi-target regulators of cellular signaling, redox homeostasis, mitochondrial function, and metabolic pathways involved in skin disease pathogenesis. Improved understanding of their molecular mechanisms, together with efforts to overcome current translational limitations, will be essential for determining their clinical potential and supporting the development of effective polyphenol-based therapeutic strategies for skin diseases.

## Figures and Tables

**Table 1:** Polyphenol-mediated regulation of cellular signaling and mitochondrial function in skin diseases.

Polyphenol	Major Targets	Signaling Pathways	Mitochondrial/Redox Effects	Biological Effects	Disease Context	References
Quercetin	PI3K/Akt, MAPK/ERK, Nrf2, NF-κB, JAK2/PKCδ	PI3K/Akt, MAPK/ERK, Nrf2/ARE, NF-κB	↓ oxidative stress; Nrf2 activation; regulation of apoptosis	Antiproliferative, antioxidant, anti-inflammatory, photoprotective	Melanoma, photoaging, UV-induced skin damage	[6,16,29,30,39,49,50]
Resveratrol	AMPK, FOXO3, Nrf2, NF-κB	AMPK/FOXO3, Nrf2/ARE, NF-κB	Regulation of ROS, mitochondrial function, and autophagy	Metabolic adaptation, antioxidant, anti-inflammatory, photoprotective	Photoaging, inflammatory skin diseases	[6,16,32,33,41,51]
Curcumin	NF-κB, MAPK, apoptosis-related proteins	NF-κB, MAPK, apoptosis-associated signaling	ROS modulation and apoptosis	Anti-inflammatory, antioxidant, antiproliferative, pro-apoptotic	Melanoma, psoriasis	[6,16]
EGCG	AMPK, PI3K/Akt, mTOR, redox regulators	AMPK/mTOR, PI3K/Akt/mTOR, antioxidant signaling	↓ oxidative damage; regulation of apoptosis and autophagy	Antioxidant, photoprotective, antiproliferative, pro-apoptotic	Photoaging, melanoma	[6,16,38,40,48]
Genistein	ROS- and apoptosis-related regulators	Oxidative stress- and apoptosis-associated signaling	ROS modulation and caspase-dependent apoptosis	Antiproliferative, pro-apoptotic, enhancement of therapeutic response	Cutaneous melanoma	[43,52]
Apigenin	Sulfiredoxin, MAPK	MAPK signaling	Regulation of oxidative-stress-associated apoptosis	Antiproliferative, anti-migratory, pro-apoptotic	Cutaneous squamous cell carcinoma	[44]
Luteolin	PI3K/Akt, Src/STAT3, ERK, NF-κB	PI3K/Akt, Src/STAT3, MAPK/ERK, NF-κB	Regulation of oxidative stress and apoptosis	Antiproliferative, anti-migratory, pro-apoptotic, anti-inflammatory	Melanoma, psoriasis	[29,31,34,35]
Kaempferol	ERK1/2, HO-1, ROS	ERK-associated antioxidant signaling	↓ ROS and oxidative-stress-induced apoptosis	Antioxidant, cytoprotective, regulation of melanocyte homeostasis	Oxidative skin damage, pigmentation-related skin disorders	[47]
Butein	TWEAK, FN14, NF-κB, STAT3	TWEAK/FN14–NF-κB/STAT3 signaling	Apoptosis-associated effects	Antiproliferative, anti-migratory, pro-apoptotic, anti-inflammatory	Cutaneous squamous cell carcinoma	[45]

↓, decrease or downregulation.

**Table 2:** Mechanistic summary of polyphenol-mediated regulation in skin diseases.

Mechanism	Key Molecular Targets	Associated Pathways	Cellular Effects	Relevance to Skin Diseases	References
Oxidative stress regulation	ROS, antioxidant enzymes (e.g., SOD, catalase)	MAPK, NF-κB, Nrf2	↓ ROS levels, ↑ antioxidant defense, improved redox balance	Inflammatory skin diseases, photoaging, chronic wounds	[6,20,21,37,38,41,42,46,48,54]
Inflammatory signaling	NF-κB, cytokines (e.g., TNF-α, IL-6)	NF-κB pathway	↓ cytokine production, ↓ inflammation	Psoriasis, atopic dermatitis	[2,3,6,21,34,53,54,55]
Cell survival signaling	Akt, ERK	PI3K/Akt, MAPK/ERK	↓ proliferation, ↑ apoptosis	Skin cancer	[4,6,13,24,29,30,31,43,44,45,52]
Metabolic stress regulation	AMPK, metabolic enzymes	AMPK pathway	Energy homeostasis, stress adaptation	Skin cancer, photoaging	[6,13,32,33,41]
Mitochondrial ROS modulation	Mitochondrial ROS, ETC complexes	Redox signaling pathways	↓ oxidative damage, redox homeostasis	Inflammatory skin diseases, photoaging	[6,19,36,37,38,39,40,41,42,48]
Mitochondrial metabolism	OXPHOS, ATP production	AMPK, metabolic pathways	Metabolic reprogramming	Skin cancer, photoaging	[13,15,19,32,36,41]
Mitochondria-dependent apoptosis	Bcl-2 family proteins, caspases	Intrinsic apoptotic pathway	↑ apoptosis	Skin cancer	[6,16,25,42,43,44,45]
Wound healing and tissue repair	ROS, inflammatory mediators, angiogenic and ECM-related factors	Nrf2, NF-κB, Notch, TGF-β-associated signaling	↓ oxidative stress and inflammation, ↑ angiogenesis and re-epithelialization, ECM remodeling	Chronic wounds, diabetic foot ulcers	[54,55,56,57,58,59,60]
Crosstalk between oxidative stress and inflammation	ROS, NF-κB, Nrf2	NF-κB, MAPK, Nrf2	Disruption of oxidative stress–inflammation feedback loop	Chronic inflammatory skin diseases	[6,21,34,53,54,55]

↑, increase; ↓, decrease.

## Data Availability

No new data were created or analyzed in this study. Data sharing is not applicable to this article.

## Abbreviations

ACC	Acetyl-CoA carboxylase
Akt	Protein kinase B
AMPK	AMP-activated protein kinase
ARE	Antioxidant response element
ATP	Adenosine triphosphate
CAT	Catalase
COX-2	Cyclooxygenase-2
ECM	Extracellular matrix
EGCG	Epigallocatechin gallate
ERK	Extracellular signal-regulated kinase
FOXO	Forkhead box O
GSH	Glutathione
GSK3β	Glycogen synthase kinase 3 beta
H2AX	Histone H2A variant X
HO-1	Heme oxygenase-1
IKK	IκB kinase
IL	Interleukin
iNOS	Inducible nitric oxide synthase
JAK2	Janus kinase 2
JNK	c-Jun N-terminal kinase
Keap1	Kelch-like ECH-associated protein 1
LPS	Lipopolysaccharide
MAPK	Mitogen-activated protein kinase
MMP	Matrix metalloproteinase
mtDNA	Mitochondrial DNA
mTOR	Mechanistic target of rapamycin
mTORC1	Mechanistic target of rapamycin complex 1
NF-κB	Nuclear factor kappa B
NQO1	NAD(P)H quinone oxidoreductase 1
Nrf2	Nuclear factor erythroid 2-related factor 2
OXPHOS	Oxidative phosphorylation
PI3K	Phosphoinositide 3-kinase
PINK1	PTEN-induced kinase 1
ROS	Reactive oxygen species
RTK	Receptor tyrosine kinase
SOD	Superoxide dismutase
STAT3	Signal transducer and activator of transcription 3
TGF-β	Transforming growth factor beta
TNF-α	Tumor necrosis factor alpha
TSC2	Tuberous sclerosis complex 2
UV	Ultraviolet
